# Accumulation of Arsenic Speciation and *In Vivo* Toxicity Following Oral Administration of a Chinese Patent Medicine Xiao-Er-Zhi-Bao-Wan in Rats

**DOI:** 10.3389/fphar.2017.00491

**Published:** 2017-07-25

**Authors:** Jiaoyang Luo, Xu Han, Xiaowen Dou, Lei Zhang, Shihai Yang, Meihua Yang

**Affiliations:** ^1^Institute of Medicinal Plant Development, Chinese Academy of Medical Sciences and Peking Union Medical College Beijing, China; ^2^College of Traditional Chinese Medicine, Jilin Agricultural University Changchun, China

**Keywords:** Xiao-Er-Zhi-Bao-Wan, arsenic speciation, toxicity, histopathology, blood biochemistry

## Abstract

Realgar-containing traditional Chinese medicines such as Xiao-Er-Zhi-Bao-Wan (XEZBW), have been widely used for thousands of years. However, events associated with arsenic-induced ailments have increasingly become a public concern. To address the toxicity of XEZBW, we studied the histopathology and blood biochemistry of rats exposed to XEZBW using technology like high-performance liquid chromatography-inductively coupled mass spectrometry to determine arsenic speciation. Our results demonstrated that dimethylarsinic acid (DMA) increased from 18.57 ± 7.45 to 22.74 ± 7.45 ng/g in rat kidney after oral administration for 7 and 14 days, which was 10-fold higher than the levels observed in controls. Trivalent arsenite As(III) showed a large increase on day 7 (26.99 ± 1.98 ng/g), followed by a slight decrease on day 14 (13.67 ± 6.48 ng/g). Total arsenic levels on day 7 (185.52 ± 24.56 ng/g) and day 14 (198.57 ± 26.26 ng/g) were nearly twofold higher than that in the control group (92.77 ± 14.98 ng/g). Histopathological analysis showed mild injury in the liver and kidney of rats subjected to oral administration of realgar for 14 days. As in the XEZBW groups, a mild injury in these organs was observed after administration for 14 days. This study inferred that the toxicity of arsenic was concentration- and time-dependent. The accumulation of DMA, a byproduct of choline metabolism, was responsible for inducing higher toxicity. Therefore, we concluded that measuring the levels of DMA, instead of total arsenic, might be more suitable for evaluating the toxicity of realgar-containing traditional Chinese medicines.

## Introduction

Realgar (As_4_S_4_), as one of the widely used traditional Chinese medicines (TCMs), has been prescribed in combination with other herbal medicines to treat common colds, tonsillitis, spasms, ulcers, heat stroke, and coma in China (Wang et al., 2008; Pharmacopoeia of China, 2015) and India (Kumar et al., 2006). Furthermore, in recent years, realgar, in the form of both TCMs and western medicines, has been used for the treatment of hematologic malignancies (Zhang et al., 2009; Liu et al., 2015) for adults and infants.

Arsenic is known for its toxicity, and the toxicity of realgar-containing TCMs has also been reported to lead to symptoms such as realgar-induced renal injury (Tsai et al., 2008), which has led to increase in public concern (Cooper et al., 2007; Saper et al., 2008). Furthermore, use of these traditional medicines is forbidden in the United States and in Europe due to the presence of arsenic that is much higher than the allowed limits (Liu et al., 2008a,b). To some extent, the potential toxicity of realgar not only slowed down the international adoption of TCMs, but also restricted medical innovation and development.

To date, opinions regarding the toxicity of arsenic are two-sided. One side argues that realgar should be used as a complementary and alternative medicine, and that if realgar is removed from arsenic-containing TCMs, these drugs would no longer be toxic. However, several studies have indicated that the therapeutic effects of these medicines reduce when realgar is removed (Fang et al., 2007; Fu et al., 2007). Previous studies also verified that realgar is one of the active components in An-Gong-Niu-Huang-Wan (AGNH), which helps prevent LPS-induced neuroinflammation in microglia-neuron cultures (Zhang et al., 2010, 2012). Another side of the opinion argues that the toxicity of arsenic depends on its chemical form and concentration in the biological systems (Cao et al., 2014; Contreras-Acuña et al., 2014). For example, arsenic in the form of sulfides (As_2_S_2_ or As_4_S_4_) is indiscerptible in water (only 4% is bioavailable in physiological gastric juice or intestinal fluids (Koch et al., 2007), therefore, it is much less acutely toxic than arsenite and arsenate (Lu et al., 2011a,b,c). However, it is still unclear whether realgar-containing TCMs are safe for long-term use. In addition, arsenic accumulation as related to variation in morphology of diseases has yet to be investigated.

The purpose of the present study was to investigate the accumulation of arsenic speciation in rat tissues and evaluate the *in vivo* toxicity. Since the toxicity of different forms of arsenic is variable, arsenic speciation in tissues should be determined prior to evaluating the toxicity of realgar-containing TCMs. In this study, Xiao-Er-Zhi-Bao-Wan (XEZBW), a realgar-containing TCM (3.6% realgar, w/w), which has been widely used to treat infant vomiting, diarrhea, and cold, was investigated. By using high-performance liquid chromatography-inductively coupled mass spectrometry (HPLC-ICP-MS), different arsenic speciation in liver and kidney of rats was determined. The histopathology and blood biochemistry in these rats were further investigated to supplement any elucidation of the potential relationship between the accumulation of an arsenic speciation *in vivo* and its toxicity.

## Materials and Methods

### Instrumentation and Reagents

An Agilent 7500ce ICP-MS (Palo Alto, CA, United States) with collision cell was applied for element specific detection coupled with an Agilent 1100 HPLC for chromatographic separations. A liquid chromatography (LC) system comprised of Hamilton PRP-X100 anion-exchange column (250 mm × 4.1 mm I.D., 10 μm) was used as the chromatographic system. The homogenate was prepared using T18 digital ULTRA-TURRAX (IKA, Germany). Infinite M1000 multimode microplate reader (TECAN, Switzerland) and UV-Vis spectrophotometer (Agilent, United States) were used for analyzing blood biochemistry. A TGL16M hydro-extractor was used to centrifuge samples. The optimum ICP-MS conditions were summarized as follows, RF power: 1,500 W; plasma Ar flow rate: 15 L/min; make up Ar flow rate: 1 L/min; carrier Ar flow rate: 1 L/min; H_2_ flow rate: 3.5 mL/min; He flow rate: 4 mL/min. The mobile phase was prepared using 0.025 mol/L ammonium dihydrogen phosphate solution (A) and ultrapure water (B), the forms were separated by a gradient elution program: 20–20% (v/v) B at 1.0–2.0 min; 20–70% B at 2.0–7.0 min; 70–99% B at 7.0–10.0 min; 99–20% B at 10.0–12.0 min; 20–20% B at 12.0–14.0 min.

All reagents were of at least analytical-reagent grade purity. The prepared working solutions of As(III), DMA, MMA, and As(V) were diluted from the stock solution (the concentrations of each forms were 124.3, 97.4, 46.2, and 32.4 μg/g) purchased from the National Center for Reference Materials (NCRM, Beijing, China) with ultra-pure water (18.2 MΩ cm^-1^) obtained from a Milli-Q (Millipore, United States) system. Working solutions of total arsenic were prepared by stepwise dilution of the stock solution obtained from the NCRM with nitric acid (3%, v/v). Rat aspartate aminotransferase (AST) ELISA kit and rat creatinine ELISA kit were purchased from Shanghai Weifan Biotechnology Co., Ltd. (Shanghai, China). Blood urea nitrogen (BUN) kit and alanine aminotransferase (ALT) were purchased from Nanjing Jiancheng Bioengineering Institute (Nanjing, China).

### Animals

This study was carried out in accordance with the recommendations of the Institutional Animal Care and Use Guidelines in China. The protocols were approved by the Institutional Animal Care of Medicinal Plant Development, Chinese Academy of Medical Sciences. The male Sprague-Dawley rats (180–220 g) (Fang et al., 2007; Zhang et al., 2010) were purchased from the Beijing HFK Bioscience Co., Ltd. (Beijing, China). Before the experiments, the rats were kept under standard laboratory conditions (12/12 h light/darkness, 20 ± 2°C, 60 ± 5% humidity) for 1 week and fasted for 12 h with free access to water prior to the experiments.

The arsenic accumulation experiment was performed as follows. First, 33 rats were randomized into six groups: control group (three rats for day 0), low dosage XEZBW (1250 mg/kg) groups (six rats for 1 week and six rats for 2 weeks), high dosage XEZBW (3750 mg/kg) group (six rats for 2 weeks), and realgar (45.25 mg/kg) groups (six rats for 1 week and six rats for 2 weeks). Blood samples of about 0.5 mL were withdrawn from the tail of rats into heparinized polythene tubes before the animals were sacrificed. Plasma was separated by centrifugation at 5000 rpm for 5 min and stored at 4°C until analysis. The visceral organ samples were obtained after 7 and 14 days of administration, and stored at -80°C until analysis.

### Blood Biochemistry

Plasma was separated from the whole blood of rats by centrifugation at 5000 rpm for 5 min. Using multimode microplate reader and UV-V is spectrophotometer, the activities of AST, ALT, BUN, and creatinine were measured following instructions provided in test kits.

### Determination of Total Arsenic

Each accurately weighed visceral organ sample was mixed with an aliquot of 5 mL ultra-pure water, then the mixture was mashed into homogenate. To determine the amount of total arsenic, the homogenate was combined with 8 mL concentrated HNO_3_ and 2 mL concentrated HCl in pre-cleaned teflon vessels, then the sample was digested completely under the optimum parameter condition using a microwave accelerated reaction system. After digestion, the sample was diluted with HNO_3_ (3%, *v*/*v*) to 50 mL for ICP-MS [the kinetic energy discrimination model (KED) was used for arsenic detection] analysis.

### Determination of Arsenic Speciation in Visceral Organ Sample

To determine the arsenic speciation, the homogenate was vortex-mixed for 1 min and ultrasound-assisted for 10 min, then centrifuged at 15,000 ×*g* at, 4°C for 50 min. The supernatant was filtered through a 0.22 μm polytetrafluoroethylene (PTFE) millipore film before it was injected for HPLC-ICP-MS analysis.

### Histological Evaluation

Portions of the liver and kidney were removed, weighed, and fixed in 10% neutral buffered formalin. Then, the fixed tissues were paraffin embedded, processed by standard histology procedures, sectioned at 6 μm and stained with hematoxylin and eosin (H&E). Pathological assessments were done in a blind fashion. Incidence of hepatic and renal lesions was determined by histological analysis of the longitudinal midline section of the right kidney and the same portion of the liver from each animal.

### Statistical Analysis

Data was calculated as mean and standard error. The SPSS 20 software was used for statistical analysis, the significance of variation between groups in data were analyzed using a one-way analysis of variance (ANOVA), followed by Duncan’s multiple range test. The significant level was set at *p* < 0.05 in all cases.

## Results

All rats survived the entire process of oral administration of XEZBW and realgar. No apparent clinical symptoms were observed.

### Arsenic Quantification in Visceral Organ Sample

**FIGURE 1:**
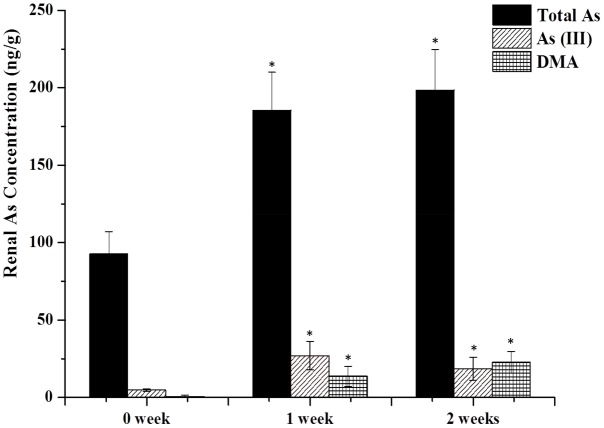
As contents in the kidney. Rats were orally given Xiao-Er-Zhi-Bao-Wan (XEZBW) 1250 g/kg for 0 days, 1 week, and 2 weeks. Tissues were collected 8 h later for analysis by high-performance liquid chromatography-inductively coupled mass spectrometry (HPLC-ICP-MS). Data are mean ± SE of six rats and expressed as ng As/g wet tissue. ^∗^*p* < 0.05, significant compared with the control group.

To delineate the potential hepatotoxicity and nephrotoxicity, arsenic contents in kidney and liver were first determined by HPLC-ICP-MS. **Figure 1** shows arsenic speciation accumulation in the kidney 2 h after the last oral dose.

Renal total arsenic contents in 1 week (185.52 ± 24.56 ng/g) and 2 weeks of administration (198.57 ± 26.26 ng/g) were nearly twofold higher than the control group (92.77 ± 14.98 ng/g). No pentavalent arsenate As(V) and monomethylarsononous acid (MMA) forms were detected in either the treatment or the control groups. In the control group, trivalent arsenite As(III) was observed (4.78 ± 1.98 ng/g) while dimethylarsinic acid (DMA) was barely present. In treatment groups, DMA increased to 18.57 ± 7.45 ng/g after 1 week, and continued to increase to 22.74 ± 7.45 ng/g at 2 weeks. As(III) also increased to 26.99 ± 1.98 ng/g after 1 week while decreased to 13.67 ± 6.48 ng/g at 2 weeks.

**FIGURE 2:**
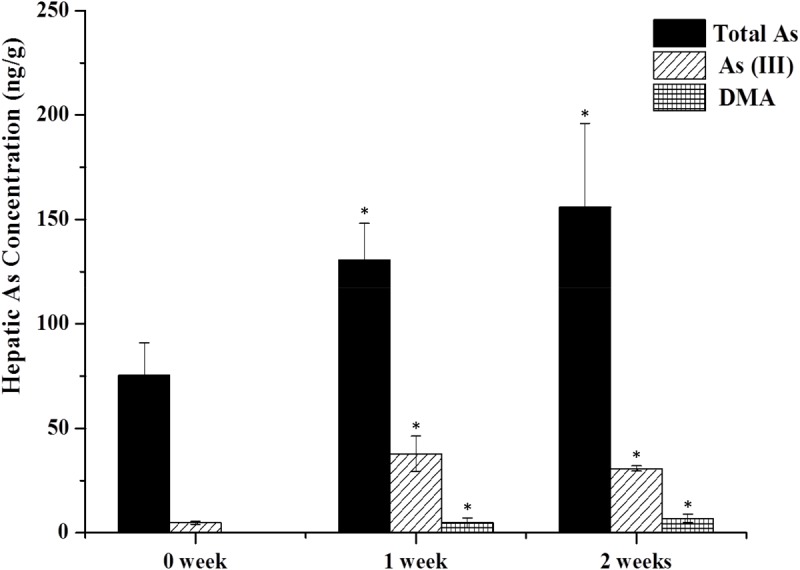
As contents in the liver. Rats were orally given XEZBW 1250 g/kg for 0 days, 1 week, and 2 weeks. Tissues were collected 8 h later for analysis by HPLC-ICP-MS. Data are mean ± SE of six rats and expressed as ng As/g wet tissue. ^∗^*p* < 0.05, significant compared with the control group.

As shown in **Figure 2**, arsenic speciation accumulated in the liver 2 h after the last oral administration and total arsenic level were also increased (twofold over controls). No trace of As(V) and MMA forms were detected in either the treatment or the control group. In the control group, no DMA was detected and the content of As(III) was 4.78 ± 0.63 ng/g. However, after 1 week of treatment, As(III) and DMA increased to 37.77 ± 8.75 and 4.77 ± 2.45 ng/g, respectively. Total arsenic level also increased to 130.56 ± 17.51 ng/g. After treatment for 2 weeks, DMA showed a gradual increase (6.87 ± 2.08 ng/g), however, As(III) and total arsenic exhibited a slight decrease (30.67 ± 1.23 and 155.91 ± 40.21 ng/g) compared to results after 1 week of treatment.

### Blood Biochemistry

**FIGURE 3:**
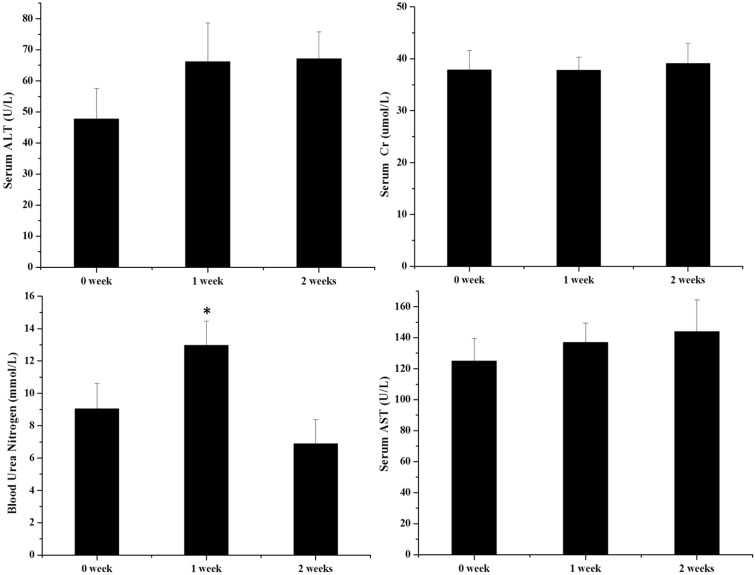
Serum activities of alanine aminotransferase (ALT), serum creatinine, blood urea nitrogen (BUN), and aspartate transaminase (AST). Rats were orally given XEZBW 1250 mg/kg for 0 days, 1 week, and 2 weeks. Blood was collected 8 h later for analysis. Data are mean ± SE of six rats. ^∗^*p* < 0.05, significant compared with the control group.

To evaluate the potential hepatotoxicity and nephrotoxicity of XEZBW, we assayed biomarkers such as BUN, creatinine, AST and serum ALT, and the results were illustrated in **Figure 3**. ALT and AST are well-known markers of liver injury. In this study, no significant change in ALT and AST (*p* > 0.05) was observed after oral administration of 1250 mg/kg XEZBW (66.20 ± 12.40 U/L and 136.90 ± 12.60 U/L on day 7; 67.17 ± 8.65 U/L and 143.99 ± 20.39 U/L, on day 14) compared to control group (47.72 ± 9.86 U/L and 124.96 ± 14.67 U/L). BUN and creatinine are biomarkers of renal injury. **Figure 3** showed that on day 7 after oral administration of 1250 mg/kg XEZBW, BUN was significantly increased (12.98 ± 1.50 mmol/L), followed by a decrease on day 14 (6.87 ± 1.51 mmol/L) compared to control group (9.03 ± 1.67 mmol/L). Meanwhile, no significant change in creatinine (*p* > 0.05) after both 1 week (37.83 ± 2.45 μmol/L) and 2 week administration (39.12 ± 3.84 μmol/L) compared to control group (37.86 ± 3.72 μmol/L) was observed.

### Histopathology

**FIGURE 4:**
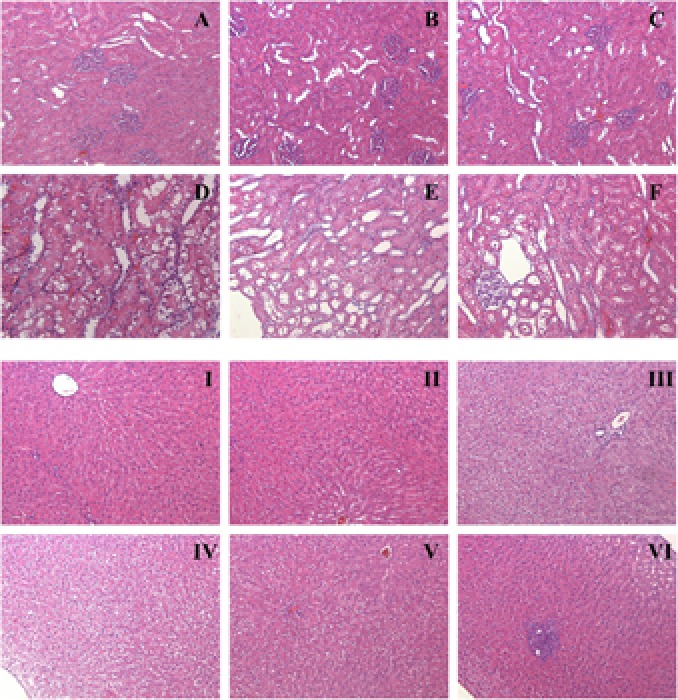
Representative histopathology of kidney **(A–F)** and liver (**I–VI**). Rats were orally given XEZBW (1250 mg/kg) for 1 week (**B** and **II**) and 2 weeks (**C** and **III**), h-XEZBW (3750 mg/kg) for 2 weeks (**D** and **IV**), realgar (45.25 mg/kg) for 1 week (**E** and **V**) and 2 weeks (**F** and **VI**) or distilled water (**A** and **I**). Tissues were fixed in formalin and stained with hematoxylin and eosin (H&E). Magnitude (100×).

Results of histopathological analysis are shown in **Figure 4**. Kidneys from the XEZBW 1 week group exhibited nearly normal appearance; the glomeruli were intact, while proximal tubular cells were slightly swollen, foci of tubular vacuolation and degeneration were observed. Renal tubular damage after oral administration of h-XEZBW (threefolds over clinical dose) was the most severe among all groups.

After oral administration for 2 weeks (including both XEZBW and h-XEZBW groups), foci of blood cysts from tubular necrosis were observed. In **Figure 4C** (oral administration of XEZBW for 2 weeks), mild impairment of nephrocytes and inflammatory cellular infiltration were observed. However, these phenomena were not observed in **Figure 4B** (oral administration of XEZBW for 1 week). The amounts of total arsenic and DMA in the 2 week group were not larger than those in the 1 week group, which suggests that the levels of total arsenic and DMA in these groups may be the minimal toxic dose for inducing cytopathy.

Representative histopathology photos of the liver are shown in **Figure 4**. Compared to controls, oral administration of XEZBW for 1 week did not lead to any variation in morphology. In contrast, other treatment groups exhibited varying degrees of hepatocyte swelling. Furthermore, foci of inflammation were also observed in oral administration of 45.25 mg/kg realgar for 2 weeks.

## Discussion

It is generally assumed that the amount of toxicant/metal at the target organ is responsible for toxicity (Lehman-McKeeman, 2010). In order to comprehensively evaluate the toxicity of arsenic-containing TCMs, the metabolism and accumulation of arsenic in tissues need to be investigated. Oral administration of Liu-Shen-Wan (LSW), an arsenic-containing TCM, did not lead to any significant arsenic accumulation compared to the control group in both liver and kidney. In contrast, oral administration of arsenite and arsenate led to significant arsenic accumulation. These results demonstrated that realgar-containing LSW was poorly absorbed and distributed to various tissues and therefore, did not reach a critical concentration to cause any tissue damage as compared to administration of the same amount of arsenic in the forms of arsenite and arsenate (Liu et al., 2011). Lu et al. (2011c) orally administered AGNH, realgar and arsenite to mice, and showed that the accumulation of arsenic after the administration of arsenite was approximately100-fold higher than that in realgar-treated liver (6200 vs. 68 ng/g). In addition, the paper argued against the previous assumption that herbal ingredients in AGNH would reduce arsenic release and bioavailability (Tang and Wang, 2005). Taken together, the aforementioned studies have proven that realgar and realgar-containing TCMs are much less toxic than arsenite and arsenate.

In higher animals, the most common inorganic arsenic [As(V) and As(III)] (ATSDR, 2005) is metabolized by oxidative methylation to form MMA(V), which will then be reduced to MMA(III) and then to DMA(V) (Fukai et al., 2006; Thomas, 2007). Since toxicity depends on the forms of heavy metals and deleterious elements [arsenite (LD_50_), 15 mg/kg; arsenate (LD_50_), 112–175 mg/kg; MMA (LD_50_), 700 mg/kg, and DMA (LD_50_), 2600 mg/kg], the quantity of each of these forms accumulated in tissues could be an important biomarker for evaluating arsenic toxicity. In this study, the accumulation of arsenic speciation was investigated in liver and kidney, and only the amounts of As(III) and DMA were determined. Interestingly, in the kidney, DMA progressively increased, while As(III) increased initially then decreased. The change in As(III) levels was different in the kidney compared to that in the liver.

Besides, Lu et al. (2011a) evaluated the hepatotoxicity and blood biochemistry after oral administration of AGNH in mice. Their result showed that both ALT and AST levels in the AGNH group were not significantly different from the controls (68 vs. 42 U/L; 380 vs. 257 U/L). In addition, BUN levels were also not elevated after treatment with AGNH (Tang and Wang, 2005). Wei et al. (2009) analyzed rat blood after oral administration of realgar for 3 weeks, and found no significant change in ALT on days 7, 14, and 21 compared to controls (*p* > 0.05). Furthermore, BUN level was obviously higher than controls (*p* < 0.05) only on day 7. Taken together, these results suggested that oral administration of realgar-containing XEZBW led to little change in blood biochemistry. We could infer from these results that liver and kidney functions probably remained normal after XEZBW administration.

Furthermore, previous work has demonstrated that mercury could exert harmful effect on the kidney through mercury-binding proteins, which are filtered by glomerulus (Pelclova et al., 2002). Pysher et al. (2007) determined that arsenic induces pathological damages in kidney through increasing hexokinase II expression in the renal glomerulus. Our results showed that oral administration of realgar and XEZBW for 2 weeks also induced glomerulus injury.

Comparing the arsenic content in each group, the quantity of arsenic observed after oral administration of XEZBW for 2 weeks was not significantly higher than that of the 1 week group. However, mild toxicity was detected only after 2 weeks of administration. Therefore, we speculated that toxicity is not only related to the quantity of arsenic, but also the time of arsenic exposure. Thomas et al. (2007, 2010) suggested that the methylation of arsenicals, besides being a detox process, could also be a mechanism of toxicity. Byproducts of choline metabolism like creatine, were detected after oral administration of 1 g/kg of realgar to rats for 2 weeks (Lu et al., 2011b). Arsenite is much more toxic than DMA in terms of acute poisoning. However, the major toxicity of chronic poisoning comes from the transformation of arsenite to DMA. Therefore, as the accumulation of DMA persists, toxicity increases. In our work, the total arsenic content in the 2 week groups was nearly 2-fold higher than that of the control group; whereas, the DMA content in kidneys was almost 10-fold over that of the controls (which exhibited barely detectable amount of arsenic and DMA in kidney and liver). Other arsenic speciations were not detected. Therefore, determination of DMA content could be more valuable for evaluating the toxicity of realgar-containing TCMs. Finally, a major type of lesion observed after oral administration of realgar and XEZBW for 2 weeks could lead to hepatitis and glomerular injury. Fortunately, these toxic effects appeared to be reversible, and tissue damages were improved when realgar and XEZBW treatments were terminated.

## Conclusion

In this study, arsenic speciation concentrations were analyzed in conjunction with histopathology and blood biochemistry to assess the toxicity of XEZBW. Renal and hepatic total arsenic contents in treatment groups were nearly twofold higher than the control group, while renal DMA displayed a continuous increase. Histopathology results showed that mild injuries in liver and kidney after oral administration of realgar for 2 weeks. A much milder injury was observed in kidney after oral administration of XEZBW for 2 weeks. Our results suggested that the continuous accumulation (after oral administration of realgar for 2 weeks or longer) of low dose of arsenic could lead to mild kidney injury, therefore, realgar-containing medicines should not be recommended for long-term use.

## Author Contribution

JL and MY designed the study; XH, JL, and LZ performed the experiments; JL and XD analyzed the data; JL and XH wrote the paper; MY and SY revised the paper.

## Conflict of Interest Statement

The authors declare that the research was conducted in the absence of any commercial or financial relationships that could be construed as a potential conflict of interest.
